# Targeting Inflammation Driven by HMGB1

**DOI:** 10.3389/fimmu.2020.00484

**Published:** 2020-03-20

**Authors:** Huan Yang, Haichao Wang, Ulf Andersson

**Affiliations:** ^1^Bioelectronic Medicine, Feinstein Institutes for Medical Research, Manhasset, NY, United States; ^2^Molecular Medicine, Feinstein Institutes for Medical Research, Manhasset, NY, United States; ^3^Department of Women's and Children's Health, Karolinska Institute, Karolinska University Hospital, Stockholm, Sweden

**Keywords:** HMGB1, inflammation, danger signal, RAGE, TLR4, drug target

## Abstract

High mobility group box 1 (HMGB1) is a highly conserved, nuclear protein present in all cell types. It is a multi-facet protein exerting functions both inside and outside of cells. Extracellular HMGB1 has been extensively studied for its prototypical alarmin functions activating innate immunity, after being actively released from cells or passively released upon cell death. TLR4 and RAGE operate as the main HMGB1 receptors. Disulfide HMGB1 activates the TLR4 complex by binding to MD-2. The binding site is separate from that of LPS and it is now feasible to specifically interrupt HMGB1/TLR4 activation without compromising protective LPS/TLR4-dependent functions. Another important therapeutic strategy is established on the administration of HMGB1 antagonists precluding RAGE-mediated endocytosis of HMGB1 and HMGB1-bound molecules capable of activating intracellular cognate receptors. Here we summarize the role of HMGB1 in inflammation, with a focus on recent findings on its mission as a damage-associated molecular pattern molecule and as a therapeutic target in inflammatory diseases. Recently generated HMGB1-specific inhibitors for treatment of inflammatory conditions are discussed.

## Introduction

Cells are constantly challenged by sterile or infectious stimuli that may cause injury or death. The alarm system sensing danger-induced cellular stress makes use of preformed endogenous molecules termed alarmins or damage-associated molecular pattern molecules (DAMPs) for this interaction (1). They have specified intracellular missions during homeostasis, but promotes inflammation when released in response to danger signals. HMGB1 is a chromatin-binding protein that among several undertakings regulates gene transcription, but operates as a critical DAMP after being released. Excessive amounts of extracellular HMGB1 may cause tissue injury and organ dysfunction in the pathogenesis of many different diseases of both sterile and infectious origin (2–5). Important questions in studies of HMGB1 biology concern how the molecule senses and mediates danger signals during infectious and sterile inflammation. What are the most effective approaches to specifically block HMGB1-driven inflammation? Here we focus on reviewing recent findings addressing these important issues.

## How Does Extracellular HMGB1 Initiate Inflammation?

Excessive quantities of extracellular HMGB1, released after cell death or via active secretion, produce inflammation. Receptor usage causing inflammation is totally dependent on whether HMGB1 acts on its own or in complex with partner molecules. HMGB1 is prone to bind other proinflammatory molecules including DNA, RNA, histones, nucleosomes, lipopolysaccharide (LPS), SDF-1, IL-1α, IL-1β, and additional factors. These complexes act in synergy via cognate receptors to the HMGB1-partner molecules. The HMGB1 redox isoform is key when HMGB1 acts on its own as a pro-inflammatory mediator. The redox state of the 3 cysteines present in an HMGB1 molecule determines subsequent bioactivities. Nuclear HMGB1 in a quiescent cell is always in the fully reduced form with all three cysteines expressing thiol groups. The fully reduced HMGB1 released extracellularly forms a complex with the chemokine CXCL12 (SDF-1) and initiates enhanced chemotaxis via CXCR4 compared to CXCL12 acting alone (6). Gentle HMGB1 oxidation generates a disulfide bond between Cys23 and Cys45, but keeping Cys106 in the reduced form. This modification converts extracellular HMGB1 to a potent activator of pro-inflammatory cytokine production via TLR4 receptor stimulation (7). Disulfide HMGB1 loses its capacity to activate TLR4 when it is either reduced or further oxidized. The ability to bind to MD-2 is also abolished by substituting Cys 45 or Cys 106 by an alanine residue (8). Additional oxidation of HMGB1 generates a sulfonyl groups on one or several cysteines resulting in molecules without any proinflammatory capacity on its own (7). The interchange between the reduced and disulfide isoforms is reversible, while sulfonyl HMGB1 is irreversibly converted.

Even if the list of reported HMGB1 receptors is quite extensive, only two receptor systems, RAGE and TLR4, are fully confirmed to act as established HMGB1 receptors. Many of the receptor systems claimed to perform as HMGB1 receptors are actually receptors for molecules complex-bound to HMGB1. When disulfide HMGB1 activates the TLR4 complex, it binds to MD-2 which forces two TLR4 chains together to form a complex that can bind intracellular signal transduction molecules (9). The binding site for HMGB1 on the MD-2 molecule is distinct from that for LPS. The biology created by HMGB1-RAGE interactions is a fascinating story that has recently been delineated (10, 11). There are approximately 700 publications on PubMed examining HMGB1-RAGE activation. The great majority concludes that HMGB1 binding to RAGE leads to a direct NF-kB activation and subsequent cytokine formation. However, macrophages expressing both TLR4 and RAGE, do not produce cytokines when stimulated by any HMGB1 isoform if TLR4 is functionally inactivated or absent. That would not be the expected result if HMGB1-RAGE activated cytokines directly. The novel discoveries by Lu and Billiar revealed that RAGE provides a transport route for HMGB1, and above all, for HMGB1-partner molecule complexes by endocytosis to the endolysosomal compartment (11). Under the acidic conditions in the lysosome system, HMGB1 has the unique ability to act as a detergent in the lysosomal membrane. The HMGB1-transported partner molecules will thus not be degraded in the lysosomes as expected, but leak out from the permeabilized lysosomes into the cytosol to reach cognate cytoplasmic receptors that will be activated to cause inflammation (11).

HMGB1 holds two defined LPS-binding sites enabling HMGB1 to bring LPS from the extracellular space via RAGE and the lysosomal compartment to cytosolic caspase 11. TLR4 deficient mice have been shown to succumb to endotoxemia in the presence of increased levels of HMGB1, while caspase 11 gene deficient mice survived (12, 13). These results emphasize the functional importance of caspase-11 as a pathogenic LPS receptor. HMGB1 operates as an LPS-carrier necessary to enable caspase-11-mediated pyroptosis. Caspase-11 oligomerization and activation are caused by LPS lipid A binding to the CARD domain of caspase-11 (14). This activated oligomerized form of caspase-11 cuts gasdermin D and the truncated gasdermin D will subsequently generate pores in the plasma membrane resulting in secretion of IL-1α, IL-1β, and HMGB1 and may terminally cause cellular pyroptosis (15). Another fundamentally important function exerted by cleaved gasdermin D is to promote coagulation running the risk to escalate into disseminated intravascular coagulation (DIC), a life-threatening event during systemic inflammation. It has recently been demonstrated that gasdermin D-induced pores can generate enhanced cell membrane expression of rotated phosphatidylserine enabled via a calcium-dependent phospholipid scramblase (15). This process markedly promotes the pro-coagulant activity of tissue factor, a central initiator of coagulation. The HMGB1-mediated transfer of LPS to caspase-11 thus represents the initial step in the cascade culminating in DIC generation.

An analogous strategy used by extracellular LPS to reach cognate intracellular receptors has also been identified for extracellular nucleic acids (16). Extracellular DNA bound to HMGB1 can be endocytosed by cells via RAGE to reach cognate DNA receptors like endosomal TLR9 or cytoplasmic cGAS or the AIM2 inflammasome complex (16–19). This biology may have detrimental effects in flares of lupus or in response to major trauma. The HMGB1/RAGE-assisted cellular import system thus performs an important task by alerting cells about a dangerous environment. HMGB1 is an alarmin with dual functions- warning the extracellular environment about cells in distress and informing cells about a hazardous extracellular surrounding.

## Pathogenic Role of HMGB1 in Immunosuppression in Sepsis

Sepsis is attributable to both exaggerated inflammatory responses and subsequent immunosuppression (20–22). When initially secreted by innate immune cells at relatively low amounts, HMGB1 might still be pro-inflammatory during the early stages of sepsis (23). However, when it is released by the liver (11) and other somatic cells at overwhelmingly higher quantities, HMGB1 could also induce immune tolerance (24, 25), macrophage pyroptosis (10, 11), and immunosuppression (26), thereby impairing the host's ability to eradicate microbial infections (27, 28).

This notion is supported by the relative higher affinity of HMGB1 to receptors (e.g., TLR4/MD-2 complex, with a disassociation equilibrium constant of 12 nM) that are involved in the activation of innate immune cells (9), whereas HMGB1 has a relative lower binding affinity to other receptors (e.g., RAGE with a disassociation equilibrium constant of 97–710 nM) (29, 30) that are required for HMGB1 endocytosis and the resultant macrophage pyroptosis. We thus propose that upon active secretion by innate immune cells or passive release by somatic cells, extracellular HMGB1 binds TLR4 (31) to induce the expression and production of various cytokines and chemokines, but triggers macrophage pyroptosis if it binds to RAGE and is internalized via receptor-mediated endocytosis (10, 11).

As aforementioned, HMGB1 can also bind many negatively charged pathogen-associated molecular pattern molecules (PAMPs, e.g., CpG-DNA, endogenous extracellular DNA or LPS) to facilitate their cellular uptake via similar RAGE-receptor-mediated endocytosis. Consequently, HMGB1 not only augments the PAMP/DAMP-induced inflammation (16), but also promotes the PAMP/DAMP-induced pyroptosis (11), leading to dysregulated inflammatory responses as well as macrophage depletion and possible immunosuppression during sepsis. In light of our recent finding that an HMGB1-neutralizing mAb (e.g., m2G7), capable of rescuing animals from lethal sepsis and acute liver injury could also inhibit HMGB1 endocytosis (32), we propose that therapeutic strategies capable of modulating HMGB1-mediated immune over-activation and/or associated immunosuppression could be developed in the clinical management of inflammatory diseases.

## HMGB1 Antagonists of Potential Clinical Interest

Several different strategies have been shown successful in inhibiting HMGB1-dependent inflammatory processes, especially aiming at blocking TLR4-HMGB1 or RAGE-HMGB1 pathways. Anti-HMGB1 antibodies and recombinant HMGB1 box A protein have each demonstrated beneficial effects in a wide range of preclinical models of inflammatory diseases (5, 33). Here we report on selected HMGB1 antagonists with a potential of being brought to clinical trials in HMGB1-driven inflammatory diseases.

## Molecules Inhibiting Rage-Mediated Endocytosis of HMGB1 or LPS-HMGB1 Complexes

**Figure 1:**
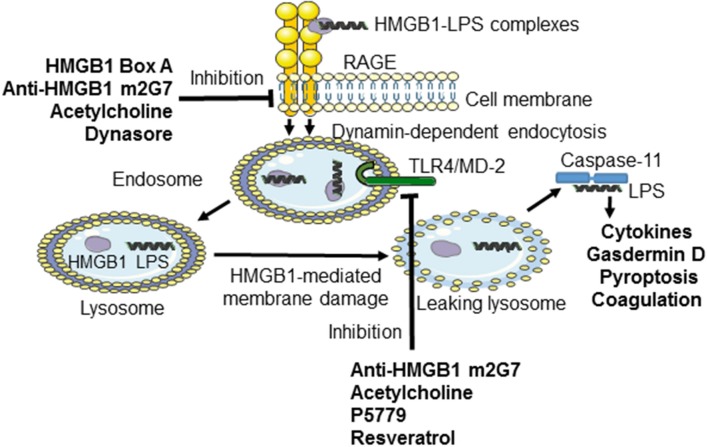
Inhibiting TLR4- or RAGE-mediated effects induced by HMGB1 or LPS-HMGB1 complexes. During endotoxemia, LPS and extracellular HMGB1 forms complexes that are endocytosed via the RAGE-dependent pathway. LPS and HMGB1 activate TLR4 system. The unique contribution by HMGB1 is disruption of the lysosomal membrane enabling LPS to reach and activate its cytosolic receptor caspase-11, which cleaves gasdermin D to form an active oligomer. Activated gasdermin D will subsequently start coagulation and cause cellular pyroptosis in murine macrophages. The HMGB1-specific inhibitors recombinant HMGB1 box A, anti-HMGB1 m2G7, and acetylcholine each inhibits the cellular internalization of LPS-HMGB1 complexes and resultant immune activation. Anti-HMGB1 m2G7 and acetylcholine also inhibit HMGB1/TLR4-mediated inflammation, whereas P5779 and resveratrol selectively block the HMGB1/TLR4 pathway only.

Previous studies established that RAGE mediates HMGB1 endocytosis via dynamin-dependent signaling (10). The concept that extracellular HMGB1-LPS complexes are imported via cell surface-expressed RAGE to the endolysosomal system from where LPS leaks out into the cytosol to activate caspase 11 has been discussed in this review (11). The study by Deng et al. also confirmed one previous report that treatment with anti-HMGB1 mAb m2G7 improves survival in experimental gram-negative sepsis (34). The observation that RAGE-mediated endocytosis of HMGB1 complexes is a pivotal event in gram-negative sepsis prompted us to study therapeutic candidate molecules with a capacity to prevent the cellular internalization of HMGB1/LPS and subsequent inflammation. We thus generated an *in vitro* assay to identify agents that inhibited RAGE-dependent import in macrophages of fluorochrome-labeled HMGB1 or fluorochrome-labeled complexes of HMGB1 and LPS (32). Our main discoveries were that m2G7, recombinant HMGB1 box A protein, acetylcholine, the nicotinic acetylcholine receptor subtype alpha 7 agonist GTS-21, and a dynamin inhibitor, all prevented cell activation and endocytosis of HMGB1, as well as of HMGB1/LPS complexes in cultured macrophages (Figure 1). The intriguing clinical therapeutic correlate to each one of these identified HMGB1 antagonists is that they can be delivered with exceptional delay (up to 24 h after sepsis initiation) with beneficial effects (35–38). This unique, and clinically important, wide therapeutic window is most likely mechanistically enabled by obstructing the HMGB1/RAGE transport route.

### HMGB1 Box A Protein

Recombinant HMGB1 box A protein has been successfully used to treat a number of experimental inflammatory models, but its mode of action has, until now, been an unresolved issue. The identification of box A-blockade of RAGE-mediated cellular import of HMGB1 and HMGB1-partner molecule complexes thus represents considerable progress, not the least because this knowledge enables an opportunity to evaluate the biological activity of individual box A batches *in vitro*. A lack of such technology has so far prevented a clinical development of box A protein. Beneficial preclinical effects by box A therapy was first reported in experimental arthritis (39), followed by CLP sepsis (35), transplantation (40), stroke (41), ischemia-reperfusion injury (42), pancreatitis (43), and acute lung injury (44).

## Molecules Inhibiting HMGB1/TLR4-Mediated Inflammation

### Peptide P5779

Macrophages that do not express TLR4 do not display nuclear NF–κB translocation or cytokine production when activated by any HMGB1 redox isoform (7). Disulfide HMGB1 subjected to cysteine mutations or redox changes loses the ability to activate TLR4. MD-2 gene-deficient macrophages do not release TNF in response to disulfide HMGB1 or any other HMGB1 isoform. Disulfide HMGB1, like LPS, binds to MD-2 with low nanomolar avidity, but to distinct MD-2 epitopes. We generated a tetramer peptide (FSSE, designated P5779) as an HMGB1 inhibitor specifically targeting the HMGB1-TLR4/MD-2 pathway (9). P5779 binds exclusively to MD-2 at the HMGB1 binding site, which preserves the responsiveness to endotoxin. The P5779 peptide does not inhibit RAGE-mediated endocytosis of HMGB1 or HMGB1/LPS complexes. P5779 (but not the scrambled control peptide) dose-dependently inhibited HMGB1-induced TNF release without affecting LPS-induced cytokine and chemokine release in primary human macrophage cultures. Therapeutic administration of P5779 protected against experimental hepatic ischemia/reperfusion-induced injury, acetaminophen-induced liver toxicity and CLP sepsis lethality (9). Furthermore, the clinical outcome of murine influenza infection was significantly improved by treatment with P5779 (45). Therapy based on P5779 administration also alleviated experimental endoluminal arterial injury-induced intimal hyperplasia and up-regulation of TLR4, HMGB1, and IL-6 expression in the affected carotid vessels. Global TLR4 gene-deficient mice demonstrated reduced inflammation and diminished HMGB1 expression after arterial injury, further supporting that HMGB1 and TLR4 are essential for vascular inflammatory responses (46). P5779 treatment conferred a striking survival advantage in an experimental pulmonary arterial hypertension model (47). Using a molecular dynamic simulation approach and surface plasmon resonance analysis, Sun et al. (48) identified that several folic acid peptides mimic the binding interaction of P5779 at the TLR4/MD-2 interaction. Addition of these P5779 mimetic peptides inhibited HMGB1-induced TNF release in cultured human macrophages. Taken together, P5779 acts as an HMGB1- inhibitor specifically targeting HMGB1-TLR4 interaction and efficiently ameliorates HMGB1/TLR4-driven inflammatory diseases (Table 1; Figure 1).

**Table 1:** Summary of efficacy of P5779 in HMGB1-driven inflammatory diseases.

**Models**	**Findings**	**References**
Acetaminophen liver toxicity in mice	Improved survival, reduced serum liver enzymes, reduced liver necrosis	(9)
Liver ischemia/reperfusion in mice	Reduced serum liver enzymes and liver inflammation	(9)
CLP-sepsis in mice	Improved survival	(9)
Arterial injury model in mice	Reduced carotid artery injury-induced intimal hyperplasia and TLR4, HMGB1, and IL-6 expression in injured vessels	(46)
Influenza in nuce	Improved survival and reduced lung edema in influenza infection	(45)
Puhnonary hypertension in rats	Improved survival in monocrotaline-induced severe pulmonar y hypertension	(47)
*In vitro*	Reduced HMGB1-induced TNF release from cultured human macrophages	(48)

### Anti-HMGB1 mAb (m2G7)

Since the development of our anti-HMGB1 m2G7 (34), many laboratories have independently confirmed the efficacy of other anti-HMGB1 mAb in many different models of sterile or infectious inflammation. The m2G7 binds to an epitope in the box A (located in HMGB1 sequence amino acids 53–63) and this binding functionally affects both HMGB1 interactions with RAGE and TLR4. Other published anti-HMGB1 mAbs have not been studied from the perspective of HMGB1 receptor inhibition and thus will not be further discussed in this section.

The m2G7 has been demonstrated to inhibit TNF production in macrophages activated by recombinant disulfide HMGB1, by HMGB1 from cultured HMGB1-transfected mammalian cells, and by HMGB1 derived from necrotic fibroblasts (7, 9). This is proof of m2G7-caused antagonistic effects on HMGB1-TLR4-mediated processes. There are many examples of preclinical HMGB1-dependent models which respond favorably to therapeutic administration of the m2G7 (Table 2). However, the inflammation is generally caused by HMGB1 activation of both TLR4 and RAGE and it is most often not possible to discriminate between the specific contributions by each receptor system. The first evidence of successful performance by the m2G7 *in vivo* came from CLP sepsis studies (34), when m2G7 therapy improved survival, a result which was confirmed in the recent report by Deng et al. (11). Systemic HMGB1 levels are increased during the acute stage of sepsis, but persistently elevated for weeks or months in both mice and patients for unknown reasons (50, 56–58). The increased HMGB1 levels post-sepsis exert a causative role for post-sepsis complications including cognitive dysfunction and anemia in the mouse CLP model. Both complications also occur after clinical sepsis, but the molecular background for this is unresolved. It is tempting to suggest HMGB1 as a cause also in the clinical situation, since HMGB1 is 99% identical in all mammals. Mice surviving CLP sepsis developed significant and persistent impairment in learning and memory, and anatomic changes in the hippocampus. Administration of the m2G7 10 days from the onset of CLP-sepsis to the survivors significantly ameliorated memory and learning disabilities, and hippocampal pathology. Systemic administration of disulfide HMGB1 reproduced the neuropathology seen after CLP sepsis (49). Systemic HMGB1 administration also caused anemia with extramedullary erythropoiesis just like CLP surviving mice. Treatment with the m2G7, provided post the acute CLP-sepsis stage, prevented the development of anemia in sepsis survivors in mice (50).

**Table 2:** Summary of efficacy of anti-HMGB1 m2G7 in HMGB1-driven inflammatory diseases.

**Models/species**	**Findings**	**References**
**Infectious diseases**		
CLP sepsis or endotoxemia in mice	Reduced lethalit y in CLP-induced sepsis and in endotoxemia	(11, 34)
CLP sepsis-survivors in mice	Reduced sepsis-induced memory impairments and brain pathology in survivors	(49)
CLP sepsis-survivors in mice	Ameliorated sepsis-induced development of anemia and stress erythropoiesis	(49, 50)
**Sterile injury**		
Islet transplantation in diabetic mice	Improved islet viability and reduced transplantation-induced inflammation	(51)
Chronic arthritis in mice	Ameliorated clinical arthritis scores, partially prevented joint destruction	(52)
Arthritis pain in mice	Ameliorated pain-like behavior in collagen antibody induced arthritis	(53)
Acetaminophen (APAP)-induced liver toxicity in mice	Attenuated APAP-induced release of ALT, microRNA-122, and abrogated inflammation	(9, 54)
Autoimmune myocarditis in mice	Reduced cardiac inflammation	(55)
Puhnonary hypertension in rats	Improved survival in monocrotaline-induced severe puhnonary hypertension	(47)

Multiple preclinical inflammatory sterile injury models likewise respond positively to m2G7 therapy. Improved islet viability and reduced inflammation after syngeneic islet graft transplantation in diabetic mice were observed in response to systemic m2G7 therapy (51). Collagen-induced arthritis and a spontaneous arthritis model were both ameliorated by m2G7 treatment. Joint destruction was prevented and clinical arthritis scores improved (52). Intrathecal m2G7 injection reversed collagen antibody-induced arthritis-induced chronic pain reactions (53). HMGB1 is an important down-stream mediator in the pathogenesis of acetaminophen intoxication and causes serious liver damage. Treatment with m2G7 significantly inhibited acetaminophen-induced release of hepatic enzymes, pro-inflammatory cytokines, and improved survival in mouse studies (9). Lundback et al. (54) confirmed these experimental results and demonstrated that administration of a humanized version of the m2G7 significantly attenuated acetaminophen-induced elevation of microRNA-122, a liver-specific microRNA, and serum levels of TNF, MCP-1, and CXCL1. Likewise, survival in experimental pulmonary hypertension in rats was significantly enhanced after m2G7 treatment (47). Systemic, as well as cardiac, HMGB1 levels are increased in mice with troponin-induced experimental autoimmune myocarditis and m2G7-based therapy reduced the cardiac inflammation and HMGB1 expression (55) (Table 2).

There are also reported therapeutic failures with the m2G7 in preclinical trials. Administration of m2G7 in a mouse model of amyotrophic lateral sclerosis showed overall very limited efficacy (59).

Treatment with m2G7 did not affect lupus nephritis in MRL/lpr mice, despite the fact that systemic levels of HMGB1 are increased in lupus (60). Successful therapeutic outcome has in contrast been reported in another mouse lupus model using a different anti-HMGB1 mAb (61).

### Resveratrol

Resveratrol is a phytoalexin phenol molecule acting as a protective endogenous antibiotic when produced in plants under stress. Resveratrol also reduces LPS-induced levels of HMGB1, IL-6, NO, and TNF in RAW 264.7 cell cultures. This TLR4-dependent process was downregulated by resveratrol-mediated inhibition of TLR4 expression (62). Resveratrol, markedly inhibited microglia activation and display of TLR4, HMGB1, MyD88, and NF-κB in the brain cortex in an experimental subarachnoid hemorrhage model (63). Furthermore, resveratrol demonstrated similar neuroprotective and anti-inflammatory effects in a neonatal hypoxic-ischemic brain injury model. Mechanistic *in vitro* and *in vivo* studies indicated that resveratrol activated SIRT1 to reduce HMGB1/TLR4/MyD88/NF-κB signaling and subsequent neuroinflammatory responses (64). The compound also demonstrated beneficial effects in an asthma model by decreasing the expression of HMGB1, TLR4, MyD88, and NF-κB mRNA levels in the lung tissue and significantly decreased the thicknesses of the airway walls (65). Together, these results indicate that resveratrol ameliorates inflammation in part via inhibition of HMGB1/TLR4-mediated inflammation (Figure 1).

### Dexmedetomidine

Dexmedetomidine is a α2-adrenoceptor agonist with anti-inflammatory effects mediated via activation of the cholinergic anti-inflammatory pathway (66). Dexmedetomidine treatment in experimental endotoxemia attenuated inflammation through downregulated TLR4 expression via a α7 nicotinic acetylcholine receptor-dependent pathway (67). It is thus of great interest that acetylcholine has the capacity to functionally inhibit both the TLR4 and RAGE pathways, the major receptor HMGB1 systems (32, 67, 68).

## Additional HMGB1 Antagonists of Clinical Interest

### Anti-HMGB1 mAb #10–22

Another extensively studied anti-HMGB1 mAb has been developed by a Japanese research group (69). The antibody, termed #10–22, recognizes an epitope in the repetitive C-terminal sequence. Successful therapeutic interventions are reported in a number of experimental neuro-inflammatory conditions, including stroke (70), traumatic brain injury (71), cognitive dysfunction after traumatic brain injury (72), spinal cord injury (73), epilepsy (74, 75), blood brain barrier dysfunction after CNS ischemia (76), hemorrhage-induced brain injury (77), neuropathic pain (78–83), and neuropathic pain-related depressive behavior (84). The antibody has also demonstrated beneficial effects in severe mouse influenza models (85, 86). Taken together, these findings demonstrated impressive treatment results in severe preclinical disease models.

### Thrombomodulin

Thrombomodulin is an endothelial cell thrombin receptor that converts thrombin into an anticoagulant. Soluble thrombomodulin also binds to HMGB1 and aids the proteolytic cleavage of HMGB1 by thrombin (87). Recombinant thrombomodulin is successfully used in Japan to treat patients with disseminated intravascular coagulation in sepsis (88).

### Haptoglobin

The major task of the acute phase protein haptoglobin is to bind and eliminate extracellular hemoglobin. Haptoglobin is in addition capable of capturing extracellular HMGB1. The haptoglobin-HMGB1 binds to CD163 on macrophages activating an anti-inflammatory response mediated via IL-10 and heme-oxygenase 1 production (89). Therapeutic administration of haptoglobin improved septic shock, lung injury, and survival in a canine pneumonia model (90). Haptoglobin is approved as an adjuvant therapy for patients in Japan with trauma, burns, and transfusion-related hemolysis.

### Metformin

Metformin occupies an important role in type 2 diabetes treatment. Metformin also has an anti-inflammatory effects, although these effects are not mechanistically fully understood. Metformin inhibits nuclear HMGB1 translocation to the cytosol and thus retains HMGB1 in the nucleus after cell activation (91). Metformin also binds directly to the C-terminal domain of HMGB1 and down-regulates inflammation by counteracting the extracellular activity of HMGB1 (92). Furthermore, the compound inhibits HMGB1 release and increases survival rate of endotoxemic mice (93).

### DNA-Conjugated Beads

HMGB1 is released and present at high levels in intestinal tissue and feces in patients with chronic inflammatory bowel diseases (IBD). Several experimental IBD models have responded very well to systemic treatment with neutralizing anti-HMGB1 antibodies. HMGB1 is a well-known DNA-binding protein, which offers an opportunity to sequester HMGB1 via DNA-conjugated beads that has been studied in experimental colitis. Oral treatment with DNA-conjugated beads significantly improved outcome in two different preclinical colitis models (94).

## Conclusion

HMGB1 antagonists have been highly successful in a broad set of preclinical inflammatory disease models, corroborating HMGB1 as an appealing therapeutic target in both infectious and sterile inflammatory conditions that currently lack efficient therapy. The next step should be to translate these preclinical studies to a clinical setting. Most preclinical treatment trials have targeted extracellular HMGB1. We suggest that this strategy should also be the preferred approach in initial future clinical studies, since we need to learn more about critical intracellular functions of HMGB1 before starting therapy studies with intracellular HMGB1 antagonists. Regardless of the indication, the success of future therapy with HMGB1 antagonists will depend on an ability to accurately measure HMGB1 on standard hospital-based instruments in order to target patients expressing excessive quantities of HMGB1.

## Author Contributions

HY, HW, and UA contributed to the elaboration of the manuscript.

### Conflict of Interest

The authors declare that the research was conducted in the absence of any commercial or financial relationships that could be construed as a potential conflict of interest.
